# Supplementary material for: Cause-Specific Mortality Fraction (CSMF) of adult mortality in Butajira, South Central Ethiopia
Source: PLOS Glob Public Health. 2023 Mar 13;3(3):e0000415. doi: 10.1371/journal.pgph.0000415 (PMC10021511; doi:10.1371/journal.pgph.0000415)
Supplement: S1 Text — (DOC) [file pgph.0000415.s001.doc]

**S1 Text. Disease classifications and corresponding verbal autopsy codes**

The burden of disease worldwide falls under three essential categories: infectious and parasitic diseases, non-communicable diseases and external causes and they contribute to a significant CoD and disabilities. Infectious and parasitic diseases (VAs-01*)* happen due to disease- causing agents and are transmitted from person to person or animal to person, including conditions like acute respiratory infection, HIV/AIDS, diarrheal diseases, malaria, meningitis, pulmonary tuberculosis, and other related infectious diseases. On the other hand, non-communicable diseases (Vas-02-9) are disease conditions of long duration and progress slowly and are not transmitted from person to person, which include neoplasms, nutritional and endocrine disorders, diseases of the circulatory system, respiratory disorders, gastrointestinal disorders, renal disorders, mental and nervous system disorders and other unspecified non-communicable diseases. Besides, external causes (VAs-12) are injuries that happen due to physical damage to a person and other related conditions, which include road traffic accidents, accidental falls, accidental drowning and submersion, intentional self-harm, assault, exposure to force of nature, and other and unspecified external CoD.
