# Supplementary material for: Cause-Specific Mortality Fraction (CSMF) of adult mortality in Butajira, South Central Ethiopia
Source: PLOS Glob Public Health. 2023 Mar 13;3(3):e0000415. doi: 10.1371/journal.pgph.0000415 (PMC10021511; doi:10.1371/journal.pgph.0000415)
Supplement: Attachment — Submitted filename: Point by Point Respnse to comments PLOS PH_.doc [file pgph.0000415.s002.doc]

Date: 08/13/2022

To: Kashif Shafique

Editor, PLoS Public Health

Subject: Resubmitting revised version

We would like to thank the editor and reviewers for the very valuable comments and suggestions and for the opportunity to resubmit a revised version of the manuscript entitled "Cause-Specific Mortality Fraction (CSMF) of Adult Mortality in Butajira, South Central Ethiopia."

The comments and suggestions were of great value, and we believe that the manuscript has improved from the revision. In the below point by point response, we respond to each point raised by the editor and reviewers.

Please do not hesitate to let us know if you have any questions.

Sincere Regards,

Hailelule Aleme Yizengaw

Corresponding Author

**Point by Point Response**

**Editor's Comments**

**Comment 1:** The objective seems sound, but the introduction and discussion require tightening to more clearly situate the rationale and findings within related literature, as well as to consider the implications of findings for programming & policy.

**Response 1:** Thank you for your attention to this request. We focused on the introduction and discussion section and revised and incorporated concepts related to the existing policies and programs.

**Comment 2:** It needs careful English language editing for grammar, usage, and punctuation. A proof read by a native English speaker to improve quality is required.

**Response 2:** Thank you for your attention to this request. After addressing the reviewers’ comments, we have checked the grammar, spelling, and typological errors by mainly using the Grammarly writing application (conscious of the meaning and other differences).

**Comment 3:** Introduction needs to provide references with the current rate and figures of CSM in Ethiopia. In first paragraph, reported literature is not matching with cited reference. Last paragraph of introduction need reference.

**Response 3:** Thank you again for your valuable comment. We added recent estimates and incorporated the comments accordingly.

**Comment 4:** First paragraph of Method section should be concise with study settings. Similarly, second paragraph which explained disease classification is additional; this is not a methodology part.

**Response 4:** Thank you again for your attention to this request. The method part is concise with study settings. Besides, the disease classification information (based on the ICD-10 diseases classification approach) is omitted from the method section and incorporated as a separate supplementary file.

**Comment 5:** The authors used two methods for assigning the cause of death. Why did you use both methods? Which method was more appropriate to explain the specific cause? Just showing the results were too much descriptive.

**Response 5:** Thank you for your valuable comments. We used two methods: Physician review to determine the cause of death till April 2020 and the InterVA algorithm then after. These two methods have their own limitations. Physician-certified verbal autopsy interpretation method is limited by its slow, expensive, and inter-reviewers variations. Even though the InterVA model is cost-effective and short period of time to undertake the cause of death assignment, the procedure is inflexible. We sated these limitations in the “S**trengths and limitations of the study”** section.

**Comment 6:** The methods section does not provide details for the computing software/package of model InterVA.

**Response 6:** Thank you for valuable comment. We included the detail explanation of the InterVA model in the method section.

**Comment 7:** Authors should provide adult mortality rates by age and sex with addition of Table 1.

**Response 7:** Thank you for your valuable comment. We added a new table (table 2) to show the adult mortality rate across the age and sex dimensions.

**Comment 8:** I recommend shortened Table 2 and Table 3 by reporting only top 20 specific causes of death. Similarly, Figure 1 and Supplementary table 1 is not informative since it contains the same information that was presented in tables.

**Response 8:** Thank you for your valuable comment. We reduced the tables to the top 20 specific causes of death (based on the overall adult deaths). We maintained figure 1 and 2, since they contains different information especially regarding age specific top 10 causes and would be more explanatory than the top 20 cause lists but we removed the supplementary table 1.

**Comment 9:** Discussion section is not technically prepared. Need to be format. Results and findings are repeated as previously reported in results section.

**Response 9:** Thank you for your valuable comment. We revised the discussion section.

**Comment 10:** I think the discussion could be shortened significantly to (1) quickly summarize findings and (2) discuss the implications, including how it relates to other literature in Ethiopia or similar contexts.

**Response 10:** Thank you for your valuable comment. We summarized the findings related to other findings of similar contexts and well discussed their implications.

**Comment 11:** Some references are irrelevant. Need to be updating and should be in a single reference style, journal required format.

**Response 11:** Thank you for your valuable comment. We followed the journal format for references and revised it accordingly, and removed the unnecessary references.

**Reviewer #1 Comments**

**Comment 1:** In introduction authors build up the justification of using CSMFs without commenting on why authors choose to do analysis using CSMFs instead of cause-specific mortality rates.

**Response 1:** Thank you for your valuable comment. We justified why we used the CSMF for this study.

**Comment 2:** Authors have completely ignored to use any measures of validity for both VA methods i.e. PCVA and CCVA. Furthermore, did authors calculated average relative error (ARE) for the estimated CSMFs? If not, please do calculate and report them for all cause of death.

**Response 2:** Thank you for your valuable comments. Since we are unable to get a Gold Standard Cause of death data in Ethiopia, we couldn’t calculate the average relative error for the estimated CSMFs. There are fragmented hospital data which are not a representative sample of deaths in the population. Comparing with such data may mislead readers and the relative error estimates will not be accurate.

**Comment 3:** It should be clearly stated whether there were any modification and/ or changes occurred in the Verbal Autopsy questionnaires and its methodology during the study period from 2008 to 2020.

**Response 3:** Thank you for your valuable comment. The data from January 2008 to April 2017 and from May 2017 to April 2020 were collected using a different but very similar format (basically using the 2012 and 2014 WHO VA questionnaires).

**Comment 4:** Authors report the use of final likelihood of at least 0.4. Kindly describe the scientific rationale behind selection of this likelihood.

**Response 4:** Thank you for your valuable comment. We have described the scientific rationale behind selection of the likelihood.

**Comment 5:** For model specification, authors predefined the prevalence of HIV/AIDS & malaria to be “low”. Citing reference # 29 & 30. Contradicting this, 11th para in discussion stated higher prevalence of HIV. Please elaborate what is the true situation of the disease and use latest statistics especially in a scenario where such statistics are used to define model parameters.

**Response 5:** Thank you for your valuable comment. We revised the discussion. Moreover, reports from World Bank /WHO and the Ethiopian Government indicated that HIV/AIDS is considered low and predefined as low in the algorithm (as basic epidemiological parameters for the model). On the other hand, most HDSS-based studies indicated considerable deaths from HIV/AIDS. But the difference may be associated with the nature of the HDSS sites where they focused most on rural populations, and the surveillance is based on VA. Moreover, the trend in using ART in rural areas is low and result in a higher death rate from AIDS.

**Comment 6:** Method section stated that residual intermediate fraction was also calculated in addition to CoD. Whereas, in result section none of the tables show any un-classified cause of death or residual intermediate cause of death.

**Response 6:** Thank you for valuable comment. Since we use the top 20 cause the undetermined cases were left out. However, the overall undetermined obtained from the two analysis were reported.

**Comment 7:** To further make sense of the reported CSMF, I would highly recommend to present these results along with 95% confidence intervals.

**Response 7:** Thank you for valuable comment. We put 95% CI for each CSMF.

**Minor concerns:**

**Comment 1:** Please provide citation for “In many developing nations, cause of death data in a sample of the population can be estimated using CSMFs with a low average error.”

**Response 1:** Thank you for valuable comment. We cited the information and make some corrections.

**Comment 2:** In 3rd para of introduction, authors reported “In Ethiopia, electronic VA databases …”. Whereas, in method sections, it is stated that computer-coded verbal autopsy model (i.e. InterVA) was used in second phase between May 2017 – April 2020. Kindly rectify this statement.

**Response 2:** Thank you for valuable comment. We meant to say that InterVA used information obtained from the electronic VA database to determine the underlying cause of death. We have now edited the paragraph.

**Comment 3:** Authors stated, “It is evidenced that there is an epidemiologic transition in the least-income countries, including Ethiopia”. When authors say “it is evidenced …” then please flourish your statement with references.

**Response 3:** Thank you for valuable comment. We cited the references.

**Comment 4:** Please use consistent abbreviations for Verbal Autopsy codes throughout the manuscript (example VAs-01 vs Vas-01-9).

**Response 4:** Thank you for your valuable comment. We used intervals for many causes of death with the conditions of that disease as a cause.

**Comment 5:** Authors should explicitly mention that this secondary analysis and should direct readers with appropriate references for further description on the methodology of primary data.

**Response 5:** Thank you for valuable comment. We mentioned it.

**Comment 6:** In methods, section for “Disease classification” is un-necessarily descriptive in nature. This information can be presented in a tabular format with few examples from VA with their corresponding ICD-10 classification.

**Response 6:** Thank you for valuable comment. We managed it.

**Reviewer #2 comments**

***** Thank you for the comments and suggestions and we already address the issues in the editor’s section.**
