# Supplementary material for: Cause-Specific Mortality Fraction (CSMF) of adult mortality in Butajira, South Central Ethiopia
Source: PLOS Glob Public Health. 2023 Mar 13;3(3):e0000415. doi: 10.1371/journal.pgph.0000415 (PMC10021511; doi:10.1371/journal.pgph.0000415)
Supplement: Attachment — Submitted filename: Response to Reviewers.doc [file pgph.0000415.s003.doc]

We want to thank the editor and the reviewers again for the very valuable comments and suggestions and for the opportunity to submit a revised version of the manuscript entitled "Cause-Specific Mortality Fraction (CSMF) of Adult Mortality in Butajira, South Central Ethiopia" for the second time. We corrected all comments point by point throughout the document.

Comments and responses to **reviewer #1**

**Question 1.** Authors have clarified why results from two systems cannot be validated due to unavailability of gold standard. I would suggest to produce kappa statistics between two methods to assess whether these two systems are producing similar results.

**Response 1:** Thank you for the valuable comments and suggestions. Based on our data, we cannot perform the Kappa statistics to measure the level of agreement between the two methods since the two methods are not applied simultaneously to same individuals.  We have used the two methods at two different time periods. But, various reports have indicated that the  InterVA, and physician-certified verbal autopsy (PCVA) methods reasonably assign similar  cause of death in the different HDSS settings. For instance, in the Agincourt HDSS site-South Africa,

<https://journals.plos.org/plosmedicine/article?id=10.1371/journal.pmed.1000325> and <https://pophealthmetrics.biomedcentral.com/articles/10.1186/1478-7954-9-46>

Kilifi HDSS site- Kenya, <https://pophealthmetrics.biomedcentral.com/articles/10.1186/1478-7954-9-49>

Nouna HDSS site- Burkina Faso, <https://pubmed.ncbi.nlm.nih.gov/22574916/>

Butajira HDSS site- Ethiopia, <https://www.ncbi.nlm.nih.gov/pmc/articles/PMC2627286/>

Moreover, the InterVA is a publicly available model for measuring and evaluating cause-specific mortality, it has an integrated platform compatible with WHO verbal autopsy instruments. <http://www.byass.uk/interva/index.htm>

**Question 2.** Regarding the final likelihood of 0.4, authors claimed in response report that they have described in revised manuscript. But looking at the revised manuscript, I cannot find their explanation. Instead authors have redirected the readers by stating, "The details were discussed elsewhere[28]". I do not agree that this can be categorized as "scientific rationale".

**Response 2:**  Thank you for your attention to this request. We revised the statement as follow: "InterVA-4 generates up to three probable causes of death for each case with their assigned statistical likelihoods or indeterminate result after the completed verbal autopsy data are entered into the InterVA-4 algorithm. The sum of the likelihoods of assigned causes has a maximum value of one. If no single cause has a final probability of at least 0.4 (the 0.4 cut-off is considerably higher than the unconditional probability of any cause) or if the sum of their likelihoods was less than one, then the residual component was "indeterminate." The details were discussed elsewhere." <https://www.ncbi.nlm.nih.gov/pmc/articles/PMC3433652/>

**Question 3.** In table # 3, the total numbers of deaths for female & male in the last column do not add up with the column total. Kindly rectify this. Similarly, the total numbers of deaths for female & male do not match with column totals (age-wise) as well as a grand total. Need careful revision in this regards.

**Response 3:** Thank you for your attention to this request. We carefully revised and corrected all the given comments.

**General responses:** We thank the editor and all reviewers for the valuable comments, suggestions, and effort to increase this manuscript's quality. We intensively work on the document to correct grammatical and editorial errors in English by inviting experts in the field. The pictures are uploaded, checked, and saved using the pace image editing platform. However, we are still **happy and ready to improve the quality of the paper further.**
